# Transbaffle, Endocardial Systemic Right Ventricle Cardiac Resynchronization Therapy in D-Transposition of the Great Arteries

**DOI:** 10.1016/j.jaccas.2025.104936

**Published:** 2025-08-06

**Authors:** Nathan R. Smith, Dhaval R. Parekh, Enrique Garcia-Sayan, Kishan Dwarakanath, Wilson W. Lam

**Affiliations:** a Department of Medicine, Baylor College of Medicine, Houston, Texas, USA; b Section of Cardiology, Department of Medicine, Baylor College of Medicine, Houston, Texas, USA; c Department of Anesthesiology, Baylor College of Medicine, Houston, Texas, USA; d Division of Pediatric Cardiology, Department of Pediatrics, Baylor College of Medicine, Houston, Texas, USA

**Keywords:** atrial switch operation, cardiac resynchronization therapy, congenital heart disease, endocardial pacing, heart failure, mustard procedure, Senning procedure

## Abstract

**Background:**

Systemic right ventricular (SRV) cardiomyopathy commonly complicates d-transposition of the great arteries with previous atrial switch repair. Cardiac resynchronization therapy (CRT) is an effective therapy but may be difficult to achieve conventionally via coronary sinus (CS) lead implantation.

**Case Summary:**

A 58-year-old man with a history of d-transposition of the great arteries and prior Mustard procedure presented with progressive dyspnea and QRS duration >200 milliseconds. No suitable CS branch was identified; therefore, we delivered CRT with endocardial SRV lead implantation via a VersaCross radiofrequency wire system. The patient experienced a QRS morphology similar to native conduction and an improvement in his symptoms and cardiac indices.

**Discussion:**

Systemic endocardial pacing has previously been demonstrated to be safe and effective in patients with normal cardiac anatomy.

**Take-Home Messages:**

Endocardial SRV leads for CRT are effective alternatives to CS lead. The VersaCross system is effective in delivering a transbaffle, SRV lead.

## Visual Summary

**Figure:**
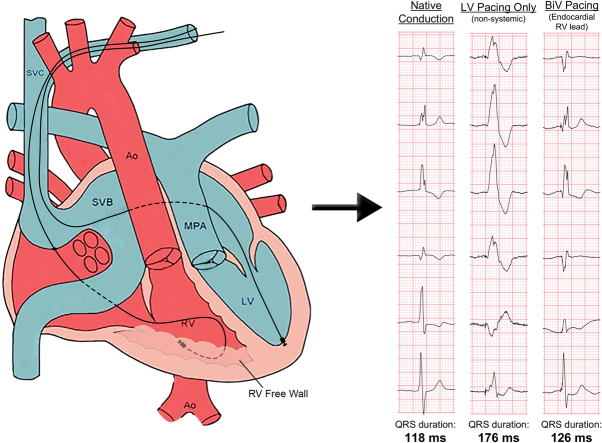

**Visual Summary:**
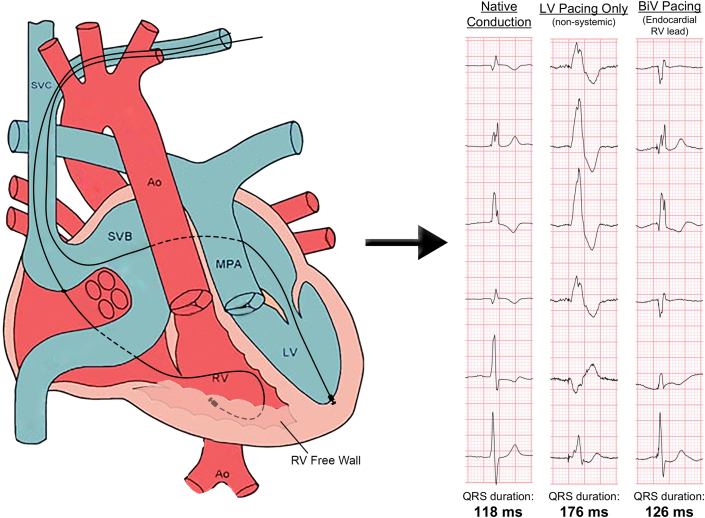
Endocardial Systemic Right Ventricular Pacing Reduces QRS Duration and Emulates Native Conduction Ao = aorta; BiV = biventricular; LV = left ventricle; MPA = main pulmonary artery; RV = right ventricle; SVB = superior venous baffle; SVC = superior vena cava.

## Case Presentation

The case was a 58-year-old man with a history of d-transposition of the great arteries (dTGA) and prior Mustard surgery, moderate-to-severe systemic tricuspid regurgitation with systemic right ventricle (SRV) cardiomyopathy, persistent atrial fibrillation status post multiple ablations on anticoagulation, and sinus node dysfunction status post dual-chamber pacemaker implanted 23 years prior who presented with progressive dyspnea. Diagnostic testing showed a QRS duration of 216 milliseconds during 52% ventricular pacing. A recent echocardiogram showed mildly reduced systolic function in the setting of severe tricuspid regurgitation (suggesting even less prograde stroke volume) and the patient's ventricular pacing burden was increasing with the QRS duration prolonged; therefore, pacing-induced cardiomyopathy became a concern. The option for cardiac resynchronization therapy (CRT) was discussed with the patient, but prior imaging had determined there was no suitable coronary sinus (CS) branch for SRV pacing, and the patient preferred not to pursue a surgical epicardial lead. A multidisciplinary team recommended CRT with endocardial SRV lead implantation.

**Take-Home Messages**

• Endocardial systemic ventricle pacing is a useful alternative to conventional CRT therapy delivered by a CS lead.
• The VersaCross system is effective in delivering a transbaffle, systemic ventricular lead.
• CRT improves intraventricular conduction delay in patients who have undergone atrial switch repair.

## Management

**Figure 1:**
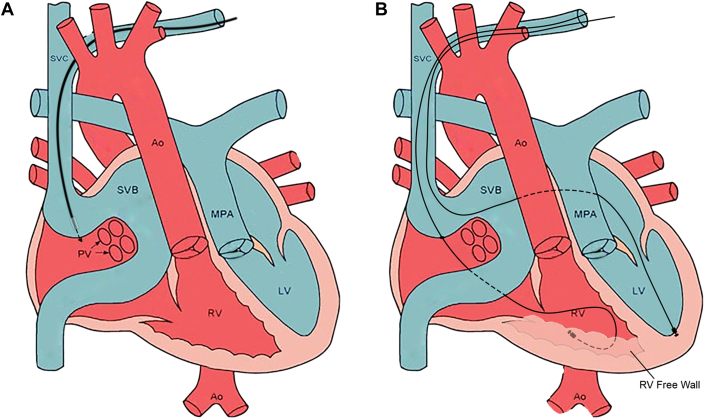
Graphical Depiction of Baffle Traversal and Implantation of the Endocardial Right Ventricular Lead (A) Depiction of Mustard anatomy with VersaCross catheter tip arriving from the SVC at the SVB wall with the catheter enclosed by its steerable sheath. (B) The VersaCross catheter has now traversed the baffle and is exchanged for a Medtronic 3830-69 Select Secure lead implanted in the right ventricular free wall. The figure also shows a left ventricular lead now implanted in the venous system. Ao = aorta; LV = left ventricle; MPA = main pulmonary artery; PV = pulmonary venous baffle; RV = right ventricle; SVB = superior venous baffle; SVC = superior vena cava. Adapted from Columbus Ohio Adult Congenital Heart Disease Program at the National Children's Hospital Heart Center, Columbus, Ohio. “Transposition of the Great Arteries After Mustard/Senning Repair” Adult Congenital Heart Association. https://www.achaheart.org/your-heart/educational-qas/types-of-heart-defects/transposition-of-the-great-arteries-after-mustardsenning-repair/ Accessed March 31, 2025.

**Figure 2:**
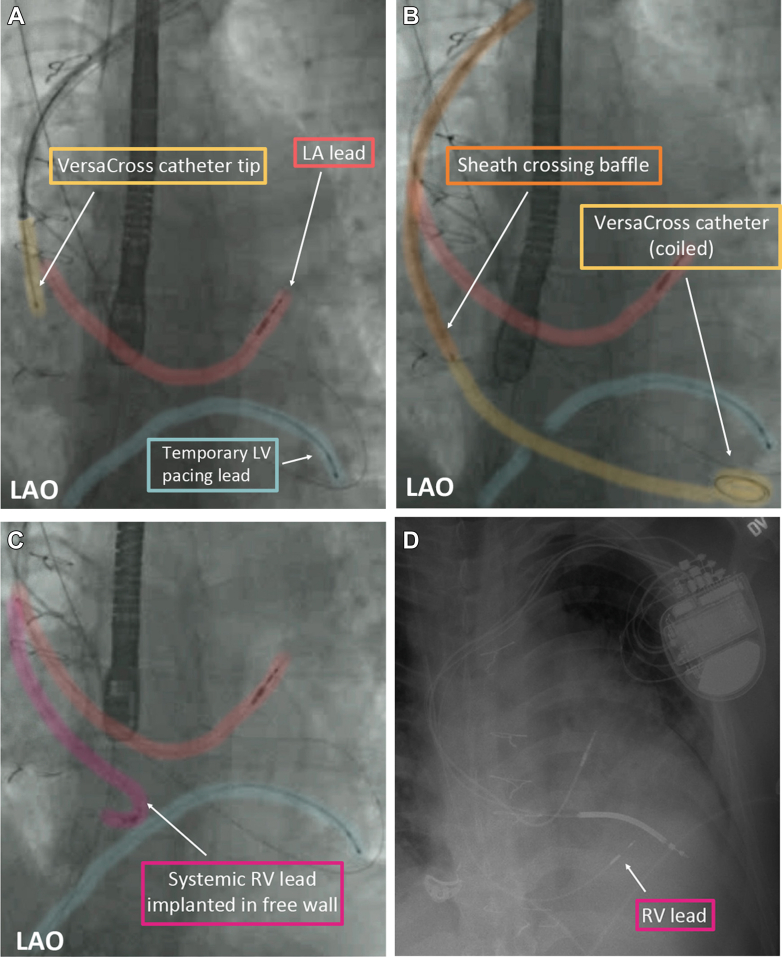
Fluoroscopic Demonstration of Baffle Traversal and Implantation of the Endocardial Right Ventricular Lead (A) Fluoroscopy of the VersaCross catheter tip abutting the superior venous baffle, preparing to traverse it (yellow). The previous implanted LA lead (red) and temporary LV pacing lead (blue) are also shown. (B) The VersaCross catheter now traverses the superior venous baffle and lies coiled in the RV apex. The sheath (orange) has been advanced to the superior venous baffle wall. (C) The systemic RV free wall lead (purple) has been placed and the VersaCross catheter and sheath have been removed. (D) Postoperative AP chest radiograph shows the LA, LV, and now RV free wall leads in place. LA = left atrial; LAO = left anterior oblique fluoroscopic view; LV = left ventricular; RV = right ventricular.

**Figure 3:**
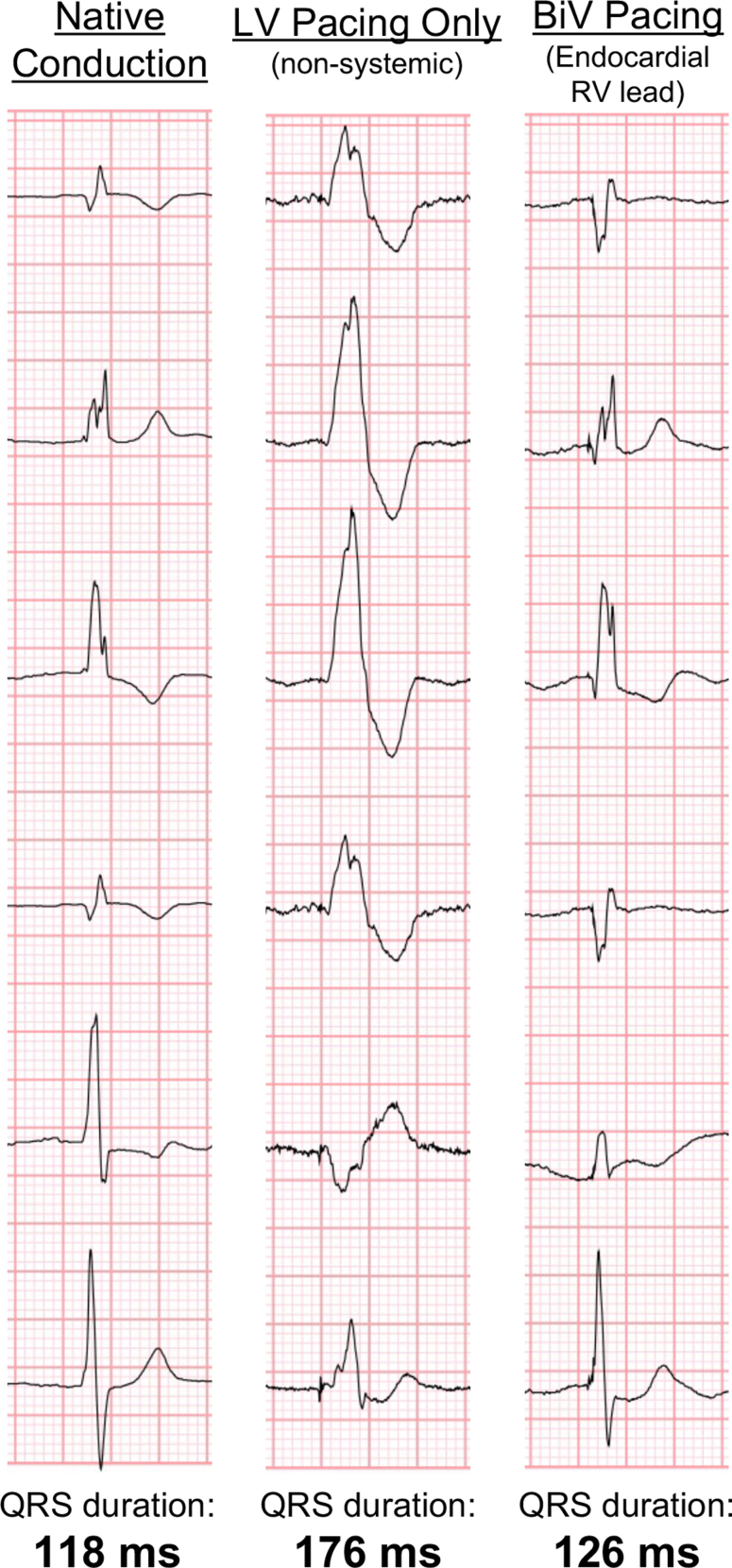
Three QRS Complexes Shown During the Patient's Intermittent Native Conduction, During Left Ventricular Only Pacing, and During Biventricular Pacing The QRS duration is significantly reduced with biventricular pacing and is morphologically similar to the patient's native conduction. BiV = biventricular; LV = left ventricular; RV = right ventricle.

While bridging with a temporary pacing wire, the previous subpulmonic left ventricular (LV) pacing lead was extracted and a single coil defibrillator lead was replaced in the left ventricle. There was no significant gradient across the superior vena cava (SVC) baffle, confirmed by venography. An appropriate location for transbaffle puncture of the SVC baffle was selected using contrast venography. Under transesophageal echocardiogram guidance, a steerable 8.5-F Agilis guide (Abbott Cardiovascular) and VersaCross radiofrequency wire system (Boston Scientific) was used to puncture the baffle. The steerable guide was exchanged for a preshaped Medtronic C315S10 guide (Medtronic). A Medtronic Select Secure 3830-69 (Medtronic) lead was then advanced to the basal anterolateral segment of the SRV (Figures 1 and 2). With biventricular pacing, the QRS duration was 115 milliseconds, comparable with the atrial-paced intrinsic conduction and junctional tachycardia (Figure 3 shows representative peri-operative EKGs).

## Discussion

SRV cardiomyopathy commonly complicates dTGA with previous atrial switch repair. Although previously state-of-the-art, the Mustard and Senning procedures have since been replaced by the arterial switch operation, which leaves the heart with a true anatomic repair in dTGA.^1^ Patients who underwent these atrial switch repair procedures are now well into adulthood, with many being affected by late complications of the procedures such as sinus node dysfunction, atrial arrhythmias, and SRV dysfunction.^2^ SRV dysfunction in particular is a leading cause of mortality in this population.^2^ CRT seems to be an effective therapy in patients with SRV cardiomyopathy but may be difficult to achieve conventionally in surgically palliated dTGA where CS anatomy is frequently not conducive to SRV lead implantation.^3^

The patient described here has several late complications of the Mustard procedure, including sinus node dysfunction, atrial fibrillation, severe tricuspid regurgitation, and SRV failure. CRT is emerging as a favorable heart failure treatment option in patients with a SRV and, given his difficult CS anatomy with a preexisting indication for anticoagulation, endocardial SRV pacing was a suitable option.

The difficulty in implanting a CS ventricular lead has led to the pursuit of suitable alternative biventricular pacing sites in typical and congenital heart disease alike. Epicardial leads are frequently used when CS anatomy is not suitable for a lead; however, this requires an often undesirable surgical approach. Conduction system pacing (CSP), including His and left bundle branch area pacing, is emerging as a favorable choice for traditional CRT. Additionally, the largest LV endocardial pacing trial to date, ALSYNC (ALternate Site Cardiac ResYNChronization), demonstrated that, in patients who had failed or were deemed unsuitable for conventional CRT, endocardial LV pacing achieved transeptally is a suitable approach and may even be more effective than epicardial lead placement.^4^

O'Connor et al^5^ previously demonstrated CSP in the atrial switch anatomy and achieved a QRS duration of 142 milliseconds, similar to our attempts at CSP through the ventricular septum in this population. The greatest barrier to reliably achieving CSP, in our experience, is reliably preshaping a suitable guide to find a suitable position for a sufficiently narrow QRS interval. Cinching a narrow QRS interval (115 milliseconds) in this patient was essential given his degree of SRV dysfunction in the setting of tricuspid regurgitation. Additionally, our patient already required a LV implantable cardioverter-defibrillator lead which transversed the SVC baffle, and we thought the introduction of a third lead through the baffle may further increase the risk of baffle stenosis. Ideally, in this scenario, a small-diameter lead capable of both CSP and defibrillation, such as the OmniaSecure lead (Medtronic), could be used but unfortunately was unavailable to us.^6^ We also considered that, in the future, the patient may benefit from cardiac contractility modulation which would require an additional 2 leads to transverse the SVC baffle and be positioned 2 cm away from any other leads in the subpulmonic left ventricle. With space in the left ventricle already limited by a hypertrophied SRV, a CSP lead would further restrict viable cardiac contractility modulation lead positions. Thus, although we contemplated CSP for this patient, we ultimately felt CRT via transbaffle, RV endocardial pacing would benefit the patient most greatly.

Akin to transseptal procedures in patients with normal cardiac anatomy, transbaffle procedures in patients who have undergone atrial switch repair are relatively common given their high burden of arrhythmias.^7^ This is typically accomplished with a Brockenbrough-style needle and sheath over a guidewire to introduce the ablation system into the atria.^8^ In this case, we used a VersaCross radiofrequency wire system to safely complete the transbaffle puncture.

The disadvantages of LV endocardial pacing in normal anatomy are increased risk of thromboembolism (necessitating lifelong anticoagulation) and the potential for iatrogenic mitral valve dysfunction. ALSYNC showed a stroke rate comparable with patients with heart failure without atrial fibrillation, and worsening mitral regurgitation was observed in only 1 of 132 patients.^4^

However, overall, cerebrovascular event rates after LV endocardial lead implantation are contested in the literature. Two systematic reviews and meta-analyses by Graham et al^9^ and Gamble et al^10^ pooled patients from the ALSYNC trial together with other published case series for a total of 362 and 384 patients, respectively. Graham et al^9^ found a statistically significant increase in the incidence of stroke (2.6 per 100 patient-years) in patients undergoing LV subendocardial lead placement compared with patients who underwent CS lead placement in large trials (0.84 per 100 patients-years and 1.5 per 100 patient-years in the Warfarin versus Aspirin in Reduced Cardiac Ejection Fraction (WARCEF) and Survival and Ventricular Enlargement (SAVE) trials, respectively), in contrast with no significant change in stroke incidence observed by Gamble et al.^10^ In patients who already have an indication for anticoagulation, such as atrial fibrillation, systemic ventricle endocardial pacing is a reasonable option, and, in our shared-decision making with the patient, we thought the benefits of systemic endocardial lead implantation outweighed the risks.

Although LV endocardial pacing is still an emerging technique, one case series suggests that LV endocardial lead extraction is safe and feasible.^11^ In particular, the lumenless lead we used (Medtronic Select Secure 3830) may successfully be removed 87% of the time with manual traction only, even when indwelling for >6 months.^12^ If our patient required lead extraction, we expect that SRV endocardial lead extraction should mirror the safety and success found in the physiological cardiac anatomy.

Two other cases previously demonstrated systemic ventricle endocardial pacing in patients who have undergone atrial switch; the first described radio frequency perforation of the baffle to introduce a systemic endocardial lead in 2009 with similar pre- and post-CRT implant QRS durations and without any prolonged follow-up.^13^ The second case was performed in a critically ill patient as a bridge for 3 months until cardiac transplant.^14^ Our case combines the VersaCross radiofrequency wire system to cross the systemic venous baffle with SRV endocardial pacing to achieve the safe delivery of CRT with a reduction in QRS duration of >100 milliseconds and a return to near-native QRS morphology while avoiding any significant adverse events in the 12 months after the procedure.

## Follow-Up

The patient was maintained on anticoagulation and had no complications at a 6-month postoperative visit. At a 1-year postoperative visit, he was euvolemic and his functional status had improved to NYHA functional class II, mostly limited by chronic back pain. His right ventricular systolic function improved to low-normal with decreased end-diastolic volume. His tricuspid regurgitation improved from moderate-severe to moderate; he may still undergo consideration for a percutaneous transbaffle TriClip implantation for repair of severe tricuspid regurgitation if this progresses again.

## Conclusions

Transbaffle, SRV endocardial CRT is a viable option in patients with dTGA with previous atrial switch repair. QRS narrowing may be better than CS or septal lead placement and avoids surgery for epicardial lead placement but should be balanced with anticoagulation to reduce thromboembolic events.

## Funding Support and Author Disclosures

The authors have reported that they have no relationships relevant to the contents of this paper to disclose.
